# The trend of schistosomiasis related bladder cancer in the lake zone, Tanzania: a retrospective review over 10 years period

**DOI:** 10.1186/s13027-023-00491-1

**Published:** 2023-02-19

**Authors:** Coletha Yohana, Jared S. Bakuza, Safari M. Kinung’hi, Bruno A. Nyundo, Peter F. Rambau

**Affiliations:** 1 Department of Natural Sciences, Mbeya University of Science and Technology (MUST), P.O Box 131, Mbeya, Tanzania; 2 Department of Biological Sciences, Dar es Salaam University College of Education (DUCE), P.O Box 2329, Dar es Salaam, Tanzania; 3 National Institute for Medical Research (NIMR), P.O Box 1462, Mwanza, Tanzania; 4 Department of Zoology and Wildlife Conservation, University of Dar Es Salaam, P.O Box 35064, Dar es Salaam, Tanzania; 5 Department of Pathology, Catholic University of Health and Allied Sciences-Bugando (CUHAS-Bugando), Box 1464, Mwanza, Tanzania

**Keywords:** Urinary schistosomiasis, Bladder cancer, Lake zone, Tanzania

## Abstract

**Introduction:**

Bladder cancer is a possible outcome of chronic urinary schistosomiasis in many endemic countries. In Tanzania, the Lake Victoria area is one of the areas with the highest prevalence of urinary schistosomiasis and higher incidences of squamous cell carcinoma (SCC) of the urinary bladder. A previous study in the area over one decade (2001–2010) showed SCC to be common in patients aged below 50 years. With various prevention and intervention programs there are likely to be notable changes in schistosomiasis-related urinary bladder cancer, which is currently unknown. Updated information on the status of SCC in this area will be useful for giving an insights into efficacy of control interventions implemented and help guide the initiation of new ones. Therefore, this study was done to determine the current trend of schistosomiasis-related bladder cancer in lake zone, Tanzania.

**Methods:**

This was a descriptive retrospective study of histologically confirmed urinary bladder cancer cases diagnosed at the Pathology Department of Bugando Medical Centre over 10 years period. The patient files and histopathology reports were retrieved and information was extracted. Data were analyzed using Chi-square and student t-test.

**Results:**

A total of 481 patients were diagnosed with urinary bladder cancer during the study period whereby, 52.6% were males and 47.4% were females. The mean age regardless of histological type of cancer was 55 ± 14.2 years. The SCC was the commonest histological type accounting for 57.0%, followed by transitional cell carcinoma 37.6%, and 5.4% were adenocarcinomas. The *Schistosoma haematobium* eggs were observed in 25.2% and were commonly associated with SCC (*p* = 0.001). Poorly differentiated cancers were observed mostly in females (58.6%) compared to males (41.4%) (*p* = 0.003). Muscular invasion of the urinary bladder by cancer was observed in 11.4% of the patients, and this was significantly higher in non-squamous than in squamous cancers (*p* = 0.034).

**Conclusion:**

Schistosomiasis-related cancers of the urinary bladder in the Lake zone of Tanzania is still a problem. *Schistosoma haematobium* eggs were associated with SCC type indicating the persistence of infection in the area. This calls for more efforts on preventive and intervention programs to reduce the burden of urinary bladder cancer in the lake zone.

## Introduction

Human schistosomiasis is one of the 21 neglected tropical diseases [16]. It is endemic in 78 countries affecting about 240 million people worldwide and 700 million people are at risk of infection [24, 50]. The disease is caused by a parasitic worm of the genus *Schistosoma* with six major species namely *Schistosoma haematobium*, *S. mansoni, S. japonicum, S. mekongi, S. intercalatum* and *S. guineensis.* While *S. haematobium* affects the urogenital system, the remaining 5 species affect the gastrointestinal tract [34, 41, 71]. Schistosomiasis is endemic in Sub-Saharan Africa, the Middle East, the Islands of Madagascar, and Mauritius [50, 71].

Chronic urinary schistosomiasis due to *S. haematobium* is commonly associated with SCC, a type of cancer affecting the urinary bladder [52, 55]. The association comes as a result of adult *S. haematobium* male and female partners living in the venous plexus of the urinary bladder in pairs. While there they mate and the female releases eggs in blood vessels, the eggs then find their way to the bladder lumen to be expelled with urine [8, 46]. However, not all eggs are expelled outside the body as some are trapped in the urinary bladder tissues [9, 12, 59]. The eggs retained in the bladder wall tissues act as a mechanical irritants releasing their antigens and provoking a strong chronic inflammatory reaction leading to the surrounding of the eggs by host immune cells contributing to granulomatous formation [9, 43, 59]. This process is also associated with the fibrotic reaction resulting in the death of *Schistosoma* eggs at the core of the fibrosis as well as the conversion of the transitional epithelium to squamous epithelium (squamous metaplasia) [9, 43]. The bladder fibrosis may further lead to bacterial infection that converts nitrite and dietary nitrates into nitrosamines which are carcinogenic [8, 51]. Thereafter, nitrosamines act on metaplastic squamous epithelium with subsequent development of SCC [8]. The SCC of the bladder is one of the most severe complications caused by chronic *S. haematobium* infection and has been reported in many parts of Africa [12, 22, 34, 41, 55, 64].

The known risk factors for urinary bladder cancer worldwide are aging, the use of tobacco products, and working with industrial chemicals [20, 21, 55]. Other reported risk factors include prolonged catheter indwelling, birth defect, diabetic medicines, obesity, chronic bladder inflammation, and inherited gene mutations [3, 60]. The aforementioned risk factors are common in developed countries causing prevalence of TCC of the bladder ranging between 90 and 95% [5, 14]. On the other hand, schistosomiasis-related SCC of the bladder is more commonly diagnosed among people with schistosomiasis in developing countries with prevalence varying from 53 to 85% [13, 53, 64]. The variation in the prevalence between SCC and TCC depend largely on the risk factors prevailing in a particular area [20, 21]. In African countries, the prevalence of *Schistosoma*-related SCC among people infected with schistosomiasis has been reported from Zimbabwe 28% and Zambia at 65% [14], Angola at 71.2% [13], South Africa at 85% [30], Ethiopia at 72.4% [62], and North-Western Nigeria at 50% [17]. The SCC incidence has been reported to decrease with the reduced prevalence of schistosomiasis [38, 42, 64]. For instance, in Egypt, SCC prevalence decreased from 75.9% in 1980 to 28.4% in 2005 which was related to control initiatives undertaken to eliminate schistosomiasis in the country [8, 27, 42, 53].

In Tanzania, bladder cancer (both SCC and TCC) has been reported from the northern and north-western parts of the country with the former being more common in both areas [37, 51]. It was observed during the studies that SCC was commonly associated with *S. haematobium* eggs detection in 46.9% and 73.5% of biopsies, respectively. This prevalence level exceeded the World Health Organization (WHO) determined threshold value [70]. Since then schistosomiasis control interventions have been undertaken at various levels aimed at reducing the prevalence and intensity of the disease [18, 54]. It is important to understand the current status of schistosomiasis-associated bladder cancer especially in the north-western part of the country. Therefore, this study was done to assess the current trend of schistosomiasis-related bladder cancer in the lake zone area of Tanzania.

## Methods

This was a descriptive retrospective study on cases of urinary bladder cancer diagnosed at the Pathology Department, Bugando Medical Centre (BMC) over 10 years period from January 2011 to May 2021. BMC is a private consultant, tertiary care facility, and teaching hospital for the Catholic University of Health and Allied Sciences Bugando (CUHAS-Bugando). It is situated along the shores of Lake Victoria in Mwanza city with a capacity of over 950 beds and serving a catchment's population of over 15 million people. This study included all histologically confirmed cancers cases of the urinary bladder diagnosed during the study period. Data were retrieved from records in the Pathology Department. Patient files kept in the medical records were also used for clinical and demographic information such as sex, age, and place of origin. Retrieved pathological information included histological type of cancer, degree of differentiation, presence of *Schistosoma* eggs, and the involvement of urinary bladder muscularis propria by cancer. Patients with incomplete data and benign confirmed cases were excluded from the study. Ethical approval for the study (No. NIMR/HQR.8a/Vol. IX/3546) was issued by the National Institute for Medical Research (NIMR) before the commencement of the study. Data were entered and analyzed using SPSS computer software version 20.0. Chi-square test was used to test for the association between categorical data while Student t-test was used for categorical and continuous data. A *p* value of ≤ 0.05 was considered to be statistically significant.

## Results

**Fig. 1:**
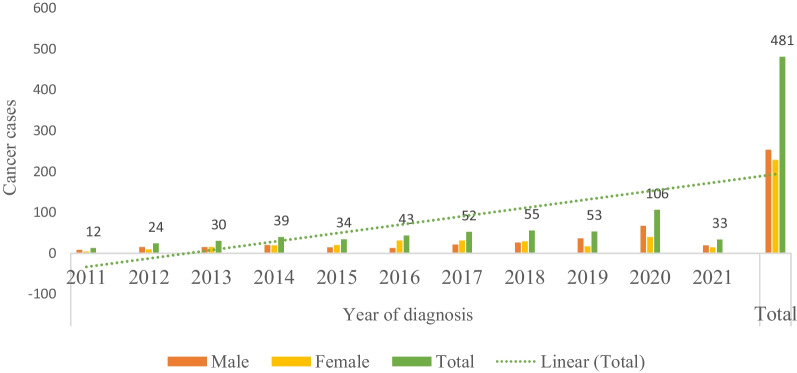
Variation of histologically confirmed cancer cases in 2011–2021

A total number of 481 patients were diagnosed with urinary bladder cancer during the study period whereby 52.6% (n = 253) were males and 47.4% (n = 228) were females, and the variation was statistically significant (*c*^2^ = 28.142, p = 0.002). The patient's age ranged from 8 to 95 years with a mean age of 55 ± 14.20 years. The mean age for males was 57.6 ± 14.941 years while for females was 53.7 ± 13.048 and the difference was statistically significant (95% CI 1.413–6.464, t (479) = 3.064, *p* = 0.002). In addition, 33.9% (n = 163) of the patients diagnosed with bladder cancer were aged below 50 years. The number of patients diagnosed with bladder cancer increased significantly (*p* = 0.002) from 2011 to 2021 (Fig. 1).

Patients whose records were studied came from different areas of Tanzania. Mwanza Region had the highest number of patients diagnosed with bladder cancer at 39.9% (n = 192), followed by Simiyu region at 17.5% (n = 84). Other regions include Shinyanga 14.8% (n = 71), Mara 8.9% (n = 43), and Geita 8.9% (n = 43). The occurrence of different types of bladder cancer varied from one region to another and the difference was statistically significant (*c*^2^ = 52.575, *p* < 0.001) (see Table 1).

**Table 1:** Variation of bladder cancer with patients' place of residence in Tanzania

Place of origin	Histological type			Total N (%)	*p* value at 95% CI
	SCC N (%)	TCC N (%)	Adenocarcinoma N (%)		
Mwanza	102 (53.1)	77 (40.1)	13 (6.8)	192 (39.9)	
Simiyu	64 (76.2)	20 (23.8)	0 (0.00)	84 (17.5)	
Shinyanga	43 (60.6)	26 (36.6)	2 (2.8)	71 (14.8)	
Mara	19 (44.2)	21 (48.8)	3 (6.98)	43 (8.9)	
Tabora	16 (64.0)	8 (32.0)	1 (4.0)	25 (5.2)	
Geita	24 (55.8)	16 (37.2)	3 (6.98)	43 (8.9)	< 0.001
Kagera	4 (26.7)	8 (53.3)	3 (20.0)	15 (3.1)	
Rukwa	2 (50.0)	2 (50.0)	0 (0.00)	4 (0.8)	
Singida	0 (0.00)	0 (0.00)	1 (100)	1 (0.2)	
Kigoma	0 (0.00)	2 (100)	0 (0.00)	2 (0.4)	
Manyara	0 (0.00)	1 (100)	0 (0.00)	1 (0.2)	
Total	274 (56.96)	181 (37.6)	26 (5.4)	481 (100)	

For all histologically diagnosed urinary bladder cancers, SCC was the most common constituting 57.0% (n = 274) of all cancer cases. Of these 44.5% (n = 122) of cancer-infected patients were males while 55.5% (n = 152) were females. SCC was significantly common in females than males (*c*^2^ = 17.703, *p* < 0.001). TCC was the second most common histological cancer being present in 37.6% (n = 181) of the patients and mostly males 64.6% (n = 117) compared to 35.4% (n = 64) in females displaying statistically significant difference (*c*^2^ = 17.706, *p* < 0.001). A total number of 26 (5.4%) patients were diagnosed with adenocarcinoma, 53.8% (n = 14) of them being males and 46.2% (n = 12) females. Among all cancers diagnosed 6.0% (n = 29) were poorly differentiated cancer, 20.8% (n = 100) were moderately differentiated and 29.3% (n = 141) were well-differentiated while 43.9% (n = 211) were not graded. Most females 58.6% (n = 17) had poorly differentiated cancer compared to males 41.4% (n = 12), and the difference was statistically significant (*c*^2^ = 14.148, *p* = 0.003). Only 11.4% (n = 55) of confirmed cancer cases had already invaded the bladder muscle. The difference in muscle invasion between males and females was not statistically significant (*c*^2^ = 0.353, *p* = 0.552) (see Table 2).

**Table 2:** Patient’s sex concerning demographic characteristics and pathological features

	Sex		*p* value at 95% CI
	Male	Female	
	N (%)	N (%)	
*Age group*			
Below 50 years	77 (47.2)	86 (52.8)	0.092
Above 50 years	176 (55.3)	142 (44.7)	
*Histological type*			
Squamous cell carcinoma	122 (44.5)	152 (55.5)	< 0.001
Transitional cell carcinoma	117 (64.6)	64 (35.4)	
Adenocarcinoma	14 (53.8)	12 (46.1)	
*Presence of Schistosoma eggs*			
Yes	62 (51.2)	59 (48.8)	0.729
No	191 (53.1)	169 (46.9)	
*Muscle invasion*			
Yes	31 (56.4)	24 (43.6)	0.552
No	222 (52.1)	204 (47.9)	
*Tumour differentiation*			
Well-differentiated	60 (42.6)	81 (57.4)	0.003
Moderately differentiated	51 (51)	49 (49)	
Poorly differentiated	12 (41.4)	17 (58.6)	
No differentiation	130 (61.6)	81 (38.4)	

*S. haematobium* eggs were detected in 121 (25.2%) of all confirmed cancer cases. Among them 79.3% (n = 96) were squamous cancers and 20.7% (n = 25) non-squamous cancers (*c*^2^ = 33.014, *p* < 0.001). The presence of *S. haematobium* eggs was not statistically different between males and females (*c*^2^ = 0.120, *p* = 0.729) (Table 2). Muscle invasion of the urinary bladder was significantly lower in *S. haematobium*-related cancers compared to those not related to the parasite (*c*^2^ = 7.268, *p* = 0.007). The mean age for patients who had *S. haematobium* eggs in cancer was 50.93 ± 15.612 while for those without *S. haematobium* eggs in cancers was 57.40 ± 13.323 and the difference was statistically significant (95% CI − 9.343 to − 3.589, t (479) = − 4.417, *p* < 0.001) (see Table 3).

**Table 3:** Histological type of urinary bladder cancer and the presence of *Schistosoma haematobium* eggs

*Schistosoma* eggs	Histological type			*c*^2^-value	*p* value at 95% CI
	SCC N (%)	TCC N (%)	Adenocarcinoma N (%)		
Present	96 (79.3)	21 (17.4)	4 (3.3)	28.402	< 0.001
Absent	180 (50.0)	158 (43.9)	22 (6.1)		
Total	274 (57.0)	181 (37.6)	26 (5.4)		

The mean age was significantly lower for squamous cancer patients (53.6 years) compared to that in non-squamous cancer patients 58.7 years (95% CI − 7.577 to − 2.514, t (479) = − 3.916, *p* < 0.001). SCC prevalence was 68.1% (n = 111) and 51.3% (n = 161) for patients below and above 50 years respectively. Muscular invasion of the urinary bladder cancer was significantly higher in non-squamous cancers than in squamous cancer (*c*^2^ = 4.500, *p* = 0.034). Poorly differentiated cancer 68.9% (n = 20) was significantly higher in patients with SCC compared to 31% (n = 9) in those with non-squamous cancers (*c*^2^ = 273.374, *p* < 0.001) (see Table 4).

**Table 4:** Variation of patients socio-demographic characteristics with squamous and non-squamous cancers

	Type of cancer		*c*^2^*-*value	*p* value
	Squamous N (%)	Non-squamous N (%)		
*Age group*				
Below fifty	111 (68.1)	52 (31.9)	12.466	0.000
Fifty and above	161 (51.3)	157 (48.7)		
*Presence of Schistosoma eggs*				
Yes	96 (79.3)	25 (20.7)	33.014	0.000
No	178 (49.4)	182 (50.6)		
*Muscle invasion*				
Yes	24 (43.6)	31 (56.4)	4.500	0.034
No	250 (58.7)	176 (41.3)		
*Tumour differentiation*				
Well-differentiated	128 (90.8)	13 (9.2)		< 0.001
Moderately differentiated	88 (88.0)	12 (12.0)	237.374	
Poorly differentiated	20 (69.0)	9 (31.0)		
No differentiation	38 (18.0)	173 (82.0)		

## Discussion

In this study, the number of patients diagnosed with bladder cancer has dramatically increased compared to the past decade with male individuals being more affected compared to their female counterparts. This is contrary to the findings reported previously in the same area [51], whereby female were diagnosed more with bladder cancer compared to male. It is not clear if this reflects an increase in incidence or just awareness of bladder cancer with a rise in detection rate. For the observed high infection in males than females, the findings concur with those reported from other areas [28, 38, 53, 73] who also found more men being infected with bladder cancer compared to females.

In the present study, bladder cancer was diagnosed in patients with more than 50 years whereby the mean age was similar to that reported by the previous study in the same study area [51]. It was found that females were likely to be diagnosed with bladder cancer at an earlier age compared to males, similar to those reported by other studies [38]. The findings are, however, different from the results reported by Rambau et al. [51], who found no difference between the sexes. However, it was observed that all bladder cancer regardless of histological types was diagnosed in 33.9% of patients with less than 50 years, indicating that in the area people of all age groups are at risk of urinary bladder cancer, and this finding corroborate with those reported in other studies in the same area [51] and from other study area [13].

Schistosomiasis-related bladder cancer is reported to occur in people of younger age compared to cancers caused by other risk factors like chemical exposure, bladder stones, and chronic indwelling catheters [3, 13, 21]. In the present study, the mean age for patients with *Schistosoma*-related cancer was statistically lower than the patients with non-*Schistosoma*-induced cancers. In addition, the mean age of patients with SCC was significantly lower than the mean age for other types of cancer, and this has previously been reported by other studies [37, 38, 51]. Furthermore, the confirmed *Schistosoma* cystitis in a patient with 8 years old has shown that schistosomiasis is acquired early in life and that *Schistosoma*-related bladder cancer develop earlier compared to non-*Schistosoma*-linked cancers as also reported by other studies [11, 72, 73].

The present study has also shown that the majority of patients diagnosed at BMC originated from Mwanza, Simiyu and Shinyanga Regions. This might have been contributed by various reasons including; firstly the fact that the three regions have been reported to be endemic for urinary schistosomiasis [6, 40], which is the known risk factor for urinary bladder cancer in developing countries including Tanzania. Another reason maybe the higher accessibility of patients to BMC for diagnosis and management.

It was observed that the number of bladder cancer patients increased significantly from 2011 to 2021 over the last decade which can be explained by various factors; firstly, the rise in the life expectancy from 47.19 years in 1971 to 65.82 years in 2020 growing at an average annual rate of 0.68% [32] has led to an increase in older patients aged 60 and above. It should be appreciated that people aged 60 and above are more at risk of bladder cancer compared to other age groups as reported previously [53]. Secondly, an increase of population in Tanzania from 35 million in 2002 to 45 million in 2012 [63], has resulted in a large number of patients suffering from various diseases including bladder cancer. Thirdly, the rise of public awareness on health issues may have resulted in increased hospital attendance for diagnosis leading to large number of bladder cancer patients observed in the present study. Fourth, the improvement of health facilities in terms of personnel and diagnostic facilities can explain the increased number of patients with bladder cancer.

The trend of urinary bladder cancer observed in this study is higher compared to that reported in the past 10 years (57% Vs 55.1%) and SCC being the predominant, similar findings have been reported in the same study area [51]. However, this is lower than those reported in Zimbabwe [64, 67], Angola [13], Yemen [2], South Africa [30] and Ethiopia [17]. Furthermore, the current SCC prevalence findings were slightly higher than those reported in north-western Nigeria [62]. Nonetheless, the trend in the present study is also above the WHO determined threshold value, suggesting that bladder cancer is still contributing to the negative health impact in Lake zone Tanzania. The observed variation in SCC prevalence between countries is contributed by various factors including; ecological variations from country to country that influences the distribution of intermediate host snails responsible for *S. haematobium* transmission [6, 46, 61, 74]. Secondly, people engaging in economic activities which involve infested freshwater contacts such as fishing, agriculture, washing and other recreational activities including swimming, wading and bathing may result in high schistosomiasis infection and eventually high SCC incidences. In addition, the implementation of schistosomiasis control programs which may result in the reduction of the disease prevalence varies from one country to another thus the observed variations in SCC incidences. Therefore, control of schistosomiasis will significantly reduce the burden of bladder cancer, especially SCC type in this area as also alluded in other reports [35].

In this study, the diagnosis of SCC was significantly higher in females than in males as also reported previously in the same study area [51]. However, the findings are different from those reported by other studies in Zimbabwe [67] and Egypt [38] where SCC prevalence was higher in males than females. This can be explained by different water contact activities performed by particular genders in respective areas. Furthermore, the differences in cultural practices influence the variation in schistosomiasis prevalence between males and females in different locations [26, 49]. The involvement of females in domestic chores and water contact increases the risk of acquiring schistosomiasis and SCC compared to males [13]. In addition, haematuria may be confused with menses in female thus delay in schistosomiasis-related SCC diagnosis and managements, leading to variation in prevalence between males and female in different countries. On the other hand, in some communities, males are working more in the rice field and fishing which may lead to high SCC incidences in males compared to females [26, 29, 38]. However, in the present study, the difference in presence of *S. haematobium* eggs between males and females was not statistically significant, signifying that both males and females are at the same risk of contracting schistosomiasis-related SCC in the Lake-zone Tanzania.

The TCC was the second most prevalent histological type observed (Table 1). This is slightly lower than the prevalence reported previously in the same area [51], as well as in Egypt [38]. Furthermore, the observed prevalence is lower than that reported in developed countries [14, 20, 53]. This suggests that there are prevailing risk factors for TCC in the country and the incidences may arise in the future due to the increase of industries and other occupational exposures [48]. The risk factors for TCC, include; the use of tobacco and occupational exposure to carcinogenic chemicals such as aromatic amines and aniline dyes [15, 20, 21]. Such risk factors are commonly reported in western countries than in developing countries, which is in line with low TCC incidences in developing countries including the one for lake zone Tanzania.

The TCC diagnosis was common in males than in female patients, this is because males are more at risk of TCC due to smoking behavior and other industrial activities compared to females [38, 53], similar findings was observed in other studies [3, 5, 25, 36, 55, 67, 68] who also reported the TCC to be the most common bladder cancer in men. However, information on patients' occupation history was missing during the present study making it difficult to make a strong argument. Furthermore, in the present study, TCC was observed to be common in patients older than 50 years indicating that exposure to TCC causing risk factors occurs late in life compared to *S. haematobium* infection which starts at young ages [3, 38, 48]. The prevalence of urinary bladder cancer has been reported to decrease in developed countries since the 1960s after the banning of tobacco use and its products [1, 53]. However, in developing countries such as Tanzania industrialization and tobacco use are now increasing which could lead to a rise in the number of patients with TCC as reported by other studies [1, 9, 48].

Histologically, a bladder cancer invading the muscularis propria is an indicator of the advanced stage which is associated with poor diagnosis [25, 53]. In this study, few cancer cases (11.4%) had invaded the bladder muscles at the time of diagnosis, contrary to previous study Rambau et al. [51] where more than half (67%) of cancers had invaded the bladder muscles. The current findings are likely a result of good accessibility to the health facility, availability of modern medical equipment such as cystoscopes and trained personnel.

In this study, only a few patients (6.0%) presented with poorly differentiated carcinoma at the time of diagnosis, and this was more common in SCC type compared to non-squamous cancers. This probably is because SCC is more aggressive than non-squamous cancers [10]. Poorly differentiated cancers were observed mostly in females than males. The poorly differentiated cancers tend to be in an advanced stage which is associated with a lower survival rate during treatment [4, 53]. Several factors are contributing to the observed differences of the advanced stage of bladder cancer between sexes; Firstly, it has been reported that most women with visible haematuria which is the common sign for urinary bladder cancer delay in seeking medical care for diagnosis, possibly due to confusion with menses [23, 57, 69]. Secondly, the thinner bladder wall in females and hormonal differences have been contributing to the variation in grades of urinary bladder cancer between sexes as discussed elsewhere [31, 36, 38, 53, 56].

Calcified *S. haematobium* eggs were seen in about a quarter of all cancer cases indicating chronic infection with the parasite, as reported by other studies [13, 19, 51, 58]. Most of the samples received for cancer diagnosis at BMC were small biopsy tissues, which could explain the observed low rate of *S. haematobium* eggs detected. The presence of *S. haematobium* eggs was highly associated with SCC type of cancer compared to non-squamous cancers implying that the former is associated with chronic urinary schistosomiasis than the latter. Similar findings have been reported by other studies [38, 51, 67]. The squamous cancer was diagnosed at equal rates in all age groups, suggesting that all residents in the Lake zone are at risk of schistosomiasis infection. On the other hand, the current study findings are contrary to the findings reported by other researchers whereby SCC was common in patients below 50 years [3, 65].

In Tanzania, only two hospital-based studies on bladder cancer have been reported [37, 51]. As also observed in the current study, both authors have reported SCC as being associated with the presence of *S. haematobium* eggs. The National Schistosomiasis and Soil-Transmitted Helminthes (STH) control program was established under the National School Health program in 2004 [33]. The program intended to control schistosomiasis and STH in school-aged children through mass distribution of praziquantel and other anti-helminthic drugs respectively. The pre-school-aged children and adults were not considered in the program which caused infected individuals to continue shedding *S. haematobium* eggs in the environment and thus perpetuates the existence of the disease [18, 24, 45].

Furthermore, in 2009 Tanzania, adopted the WHO’s schistosomiasis control initiatives through preventive chemotherapy (PCT), and the NTDs control program began the same year [33]. However, among the 10.8 million people who required preventive treatment in Tanzania in 2014, only 27% were covered [66]. Schistosomiasis control has however, contributed to reducing incidences of SCC in endemic areas [8, 27]. In Mwanza Region Tanzania for instance, some villages remained with high schistosomiasis prevalence after four rounds of MDA [45], while in Dodoma praziquantel uptake was below the WHO’s minimum levels [18]. Variation in socioeconomic, cultural, and political factors influences the participation in MDA programs in communities [24, 45]. Therefore, the factors should always be considered for successful schistosomiasis elimination in communities as recommended [24, 45, 47].

The persistence of the high prevalence of schistosomiasis-associated bladder cancer for more than two decades in Lake-Zone Tanzania shows that disease control initiatives are not successful. This could be explained several factors including; lack of health education, motivation, and commitment of the community in controlling the disease. Secondly, the consideration that the disease is of low priority caused many people not turning-up to the arranged MDA program causing the infection to remain high especially among untreated individuals in the population [7, 45, 47]. Thirdly, there is a widespread misconception that the anti-helminthic drugs are disguised birth control agents making some people avoid them [39, 44]. It is important therefore that schistosomiasis control programs are accompanied by health education for maximum impact. Early medical care-seeking behavior should be promoted to enhance the prevention of late-stage complications of urinary schistosomiasis. However, this will be possible if people are aware of the signs and symptoms of urinary schistosomiasis and its sequelae such as anemia and bladder cancer.

## Conclusion and recommendations

The number of patients infected with schistosomiasis-related urinary bladder cancer in the Lake Zone area of Tanzania has increased, with squamous cell carcinoma being predominant. This trend of schistosomiasis-related squamous cell carcinoma demand a better surveillance systems which can be implemented for control of disease and eventually elimination of health impacts caused thereof. Testing of SCC for patients presenting with schistosomiasis infection signs and symptoms should be mandatory to enable the early detection of lesions in the urinary tract system.

## Abbreviations

BMC: Bugando Medical Centre
LAP: Lower abdominal pain
LUTS: Lower urinary tract symptoms
MDA: Mass drug administration
NTD: Neglected tropical diseases
NIMR: National Institute for Medical Research
PCT: Preventive chemotherapy
SCC: Squamous cell carcinoma
STH: Soil-transmitted helminths
SPSS: Statistical package for social sciences
TCC: Transitional cell carcinoma
UTI: Urinary tract infection
WHO: World Health Organization
